# Methods for individualized assessment of absolute risk in case-control studies should be weighted carefully

**DOI:** 10.1007/s10654-016-0206-y

**Published:** 2016-10-13

**Authors:** Kevin Ten Haaf, Ewout Willem Steyerberg

**Affiliations:** Department of Public Health, Erasmus MC - University Medical Center Rotterdam, Rotterdam, The Netherlands

Risk prediction models can best be developed in prospective longitudinal cohort studies, as such studies allow optimal measurement of predictor and outcome variables and direct estimation of absolute risk [1]. Prospective cohort studies may require a large sample size or long follow-up duration for rare diseases. In contrast, case-control studies are more efficient, as they require fewer subjects and can be performed in a shorter timeframe than prospective cohort studies. However, if not explicitly nested within a cohort study, case-control studies are generally deemed less suitable for developing a risk prediction model due to their inability to allow the calculation of absolute risk [2].

Karp et al [3] propose an easily applicable method that allows the calculation of absolute risk in non-nested case-control studies. They developed a lung cancer risk prediction model based on individuals in the Montreal Lung Cancer Case-Control study and used population data from Montreal to weight the controls with age and sex strata specific study population-time. They present the model with different time horizons, and flexibility to consider predictions for various scenarios of risk factor development over time. We reflect on a number of aspects of the proposed method, in particular with regards to the weighting.

The main issue is the selection process of the cases and controls. A major limitation of case-control studies, described as early as 1959, is the difficulty to ensure that cases and controls are a representative sample of the same source population [4]. By weighting the controls with age and sex strata specific study population-time, the assumption that there are no factors influencing the selection of controls other than those considered in the weighting formula should be carefully considered. The response rates were allowed to differ by sex (males: 0.862; females 0.818), but not by age or other characteristics than age and sex. Similarly the proportion of Canadians in Montreal was set to a constant (0.954), irrespective of other characteristics. Moreover, a complete case ascertainment was assumed for the Montreal metropolitan area.

The weighting greatly affected the model intercept, which is expected, as only 1,288 controls were included in the matched case-control study from over 3 million controls in the Montreal population. Moreover, the odds ratios changed for age (from 1.1 to 3.4 per decade) and gender (from 0.7 to 1.2 for male sex). These changes in parameter estimates are also expected since the weighting is meant to correct for the age- and sex-matching of cases and controls. However, the odds ratio for the comprehensive smoking index (CSI) changed drastically as well: from 10.1 to 31.5. This illustrates that the weighting can substantially affect the parameter estimates of risk factors other than the ones used to match the cases and controls.

Finally, the authors state that the risk-stratifying performance of their method was “reasonably high”, based on assessing the range and variability of 15-year lung cancer risk across a variety of risk profiles [3]. We note that the weighting of the control series magnified the differences in risk profiles between the cases and controls substantially, as also reflected in an increase in discriminative ability (c statistic from 0.81 to 0.89). Although the weighted model apparently estimates a quite diverse range of risks, the key property for individualized risk information is calibration, which is again related to the validity of the weighting procedure. “Moderate” calibration, i.e. the observed event rate should be R% among individuals where the model predicts a risk of R%, has been suggested as the proper ambition of a proposed model [5]. To assess whether a model meets this level of calibration, the observed event rates across different risk profiles are required; i.e., the absolute risk levels need to be known. As the authors only used information from a non-nested case control study, they are unable to assess whether their method correctly estimates the absolute level of risk of an individual.

We hence agree with the authors that an external validation study of the weighted model in a population where the level of absolute risk is known is essential [3]. Such a study will be valuable in evaluating the validity of the proposed method to use non-nested case-control studies for risk model development.

## References

[1] Moons KGM, Altman DG, Reitsma JB (2015). Transparent reporting of a multivariable prediction model for individual prognosis or diagnosis (TRIPOD): explanation and elaboration the TRIPOD statement: explanation and elaboration. Ann Intern Med.

[2] Steyerberg EW (2009). Clinical prediction models: a practical approach to development, validation, and updating.

[3] Karp I, Sylvestre M-P, Abrahamowicz M, Leffondré K, Siemiatycki J (2016). Bridging the etiologic and prognostic outlooks in individualized assessment of absolute risk of an illness: application in lung cancer. Eur J Epidemiol.

[4] Mantel N, Haenszel W (1959). Statistical aspects of the analysis of data from retrospective studies of disease. J Natl Cancer Inst.

[5] Van Calster B, Nieboer D, Vergouwe Y, De Cock B, Pencina MJ, Steyerberg EW (2016). A calibration hierarchy for risk models was defined: from utopia to empirical data. J Clin Epidemiol.

